# Solution to Detect, Classify, and Report Illicit Online Marketing and Sales of Controlled Substances via Twitter: Using Machine Learning and Web Forensics to Combat Digital Opioid Access

**DOI:** 10.2196/10029

**Published:** 2018-04-27

**Authors:** Tim Mackey, Janani Kalyanam, Josh Klugman, Ella Kuzmenko, Rashmi Gupta

**Affiliations:** ^1^ Division of Infectious Disease and Global Public Health Department of Anesthesiology, School of Medicine University of California San Diego La Jolla, CA United States; ^2^ Department of Electrical and Computer Engineering Jacobs School of Engineering University of California San Diego La Jolla, CA United States; ^3^ IBM Global Business Services Washington, DC United States; ^4^ Centers for Disease Control and Prevention Atlanta, GA United States

**Keywords:** online pharmacies, drug abuse, opioid abuse, machine learning, unsupervised machine learning, prescription drug misuse

## Abstract

**Background:**

On December 6 and 7, 2017, the US Department of Health and Human Services (HHS) hosted its first Code-a-Thon event aimed at leveraging technology and data-driven solutions to help combat the opioid epidemic. The authors—an interdisciplinary team from academia, the private sector, and the US Centers for Disease Control and Prevention—participated in the Code-a-Thon as part of the prevention track.

**Objective:**

The aim of this study was to develop and deploy a methodology using machine learning to accurately detect the marketing and sale of opioids by illicit online sellers via Twitter as part of participation at the HHS Opioid Code-a-Thon event.

**Methods:**

Tweets were collected from the Twitter public application programming interface stream filtered for common prescription opioid keywords in conjunction with participation in the Code-a-Thon from November 15, 2017 to December 5, 2017. An unsupervised machine learning–based approach was developed and used during the Code-a-Thon competition (24 hours) to obtain a summary of the content of the tweets to isolate those clusters associated with illegal online marketing and sale using a biterm topic model (BTM). After isolating relevant tweets, hyperlinks associated with these tweets were reviewed to assess the characteristics of illegal online sellers.

**Results:**

We collected and analyzed 213,041 tweets over the course of the Code-a-Thon containing keywords codeine, percocet, vicodin, oxycontin, oxycodone, fentanyl, and hydrocodone. Using BTM, 0.32% (692/213,041) tweets were identified as being associated with illegal online marketing and sale of prescription opioids. After removing duplicates and dead links, we identified 34 unique “live” tweets, with 44% (15/34) directing consumers to illicit online pharmacies, 32% (11/34) linked to individual drug sellers, and 21% (7/34) used by marketing affiliates. In addition to offering the “no prescription” sale of opioids, many of these vendors also sold other controlled substances and illicit drugs.

**Conclusions:**

The results of this study are in line with prior studies that have identified social media platforms, including Twitter, as a potential conduit for supply and sale of illicit opioids. To translate these results into action, authors also developed a prototype wireframe for the purposes of detecting, classifying, and reporting illicit online pharmacy tweets selling controlled substances illegally to the US Food and Drug Administration and the US Drug Enforcement Agency. Further development of solutions based on these methods has the potential to proactively alert regulators and law enforcement agencies of illegal opioid sales, while also making the online environment safer for the public.

## Introduction

### National Opioid Crisis

It is estimated that 90 Americans die daily by overdosing on opioids, a staggering figure highlighting the human toll of this public health crisis that continues to escalate [1]. Since the year 2000, an estimated 300,000 lives have been claimed by the opioid epidemic, which has expanded beyond nonmedical use of prescription opioids into transition of use to heroin addiction and deaths occurring from illicitly manufactured synthetic opioids (such as fentanyls and their analogues [2-5]). Additionally, the US Centers for Disease Control and Prevention (CDC) estimates that the annual economic losses from this crisis equate to US $78.5 billion because of the costs of health care, addiction treatment, the criminal justice system, and lost productivity [6,7]. Rising death tolls and growing economic burden (with CDC reporting a quadrupling of deaths attributable to prescription opioids since 1999) have prompted certain state jurisdictions and President Donald Trump to declare the opioid crisis a public health emergency [8,9].

Responses to tackle the opioid epidemic have occurred at both the state and federal level, largely focused on actions aimed at reducing inappropriate prescribing, expanding opioid treatment and prevention programs (including access to the opioid antagonist and rapidly reversing overdose drug naloxone), establishing prescription drug monitoring programs, preventing drug diversion, and even the use of litigation against pharmaceutical companies [5,8,10-13]. These approaches largely fit into the five major priority areas outlined by the US Department of Health and Human Services (HHS) to combat the opioid crisis, which include improving access to treatment and recovery services, promoting use of overdose-reversing drugs, strengthening public health surveillance, enhancing research on pain and addiction, and supporting better practices for pain management. These strategic goals are being carried out through investments in science, training, various mitigation strategies, community-based activities, efforts to change prescribing and management practices, and policy making [13-15].

Seeking to further catalyze efforts around HHS’ five-part opioid strategy through technology and innovation, on December 6 and 7, 2017, the agency hosted an Opioid Code-a-Thon event that brought together over 300 participants to develop data-driven solutions to combat the opioid epidemic in three challenge tracks (treatment, usage, and prevention tracks; see Table 1) [16]. The Code-a-Thon, the first of its kind for HHS, also involved partnership with several data science providers, organizations, and platform sponsors, including Socrata, Tableau, IEEE Standards Association, and Google. The event provided participants access to a hosted data portal that included deidentified datasets from HHS, federal, state, and local governments, and the private industry (eg, datasets such as CDC WONDER—Multiple Causes of Death, Medicare Part D Prescribing Data—Centers for Medicare & Medicaid Services, National Survey on Drug Use and Health—Substance Abuse and Mental Health Services Administration, and Medical Expenditure Panel Survey—Agency for Healthcare Research and Quality), which were used by participants to build data visualizations, interpret data in new ways, build analysis tools, and propose broader solutions.

In total, over 50 teams competed and pitched their ideas through two rounds of judging, with nine selected for a final round of presentations. In the end, three winners were awarded prizes of US $10,000 each to further develop and implement their solutions [16]. The coauthors of this paper, comprising an interdisciplinary public-private team with members from UC San Diego—School of Medicine and Jacobs School of Engineering, the CDC, and IBM, participated as team Ryan Haight, as part of the prevention track (a track that asked for solutions designed to predict and analyze the supply and movement of legal and illicit opioids) and were selected as one of nine finalists (for recorded video of Code-a-Thon presentation visit the HHS YouTube video [17]). In this paper, we describe the opioid challenge we addressed, the methods and solution we used to address the challenge, the results of our analysis, and provide a discussion on our approach to move this innovation forward in an attempt to address the digital dangers of opioid abuse via social media and illicit online sellers.

### Opioid Challenge: Illicit Online Marketing of Sale of Opioids Direct-to-Consumer via Twitter

We named our Code-a-Thon team after an 18-year old adolescent from San Diego, California, who overdosed and died after purchasing the prescription opioid Vicodin from a no prescription internet seller in February 2001 [18,19]. The death of Ryan Haight eventually led to passage of federal legislation, the 2008 Ryan Haight Online Pharmacy Consumer Protection Act (RHA), aimed at curbing illegal diversion of controlled substances by making it a federal crime to purchase, sell, or import controlled substances online without a valid prescription as currently enforced by the US Drug Enforcement Agency (DEA) [18].

However, since the enactment of RHA, internet technologies have experienced rapid growth, with an estimated 84% and 65% of adult Americans using the internet and social media respectfully [20]. Furthermore, a survey conducted by the US Food and Drug Administration (FDA) of adults who had made purchases online found 23% had purchased a prescription medication online [21]. Reflecting this trend of increasing internet adoption and use of internet for health information–seeking and e-commerce, online pharmacies have also rapidly proliferated with an estimated 30,000 legitimate and illegal sites (though it is estimated that 96% of these sites fail to adhere to legal and safety requirements) in existence [22-24].

**Table 1:** Summary of US Department of Health and Human Services (HHS) Opioid Code-a-Thon challenge tracks.

Track name	Description	Winning team
Prevention track	Solutions used to better predict and analyze the supply and movement of legal and illicit opioids	Visionist Inc, solution to assess unmet need for takeback programs at pharmacies
Treatment track	Solutions to improve access to effective treatment and recovery services	Origigami Innovations, developed model for real-time tracking of overdoses
Usage track	Solutions to help identify at-risk populations and their underlying risk characteristics of opioid misuse or abuse	Visionist Inc, solution to assess unmet need for takeback programs at pharmacies

Of these tens-of-thousands of cyber-pharmacies, the internet security firm LegitScript estimates that approximately 9% sell controlled substances, with a separate report by the National Association of Boards of Pharmacy (NABP), which reviewed more than 11,000 online pharmacy websites, estimating that 13% illegally dispensed controlled substances [22,25,26]. Results from these studies also confirm findings from US government investigations, including a 2004 study by the US Government Accountability Office (GAO), where investigators were able to purchase OxyContin, Percocet, and Vicodin from no prescription online pharmacies, and a recent bipartisan report detailing how Senate investigators were able to easily purchase fentanyl online and have product shipped through the mail [27-29].

Supplementing reports from the FDA, NABP, the GAO, and Senate investigations, a number of published research studies have also found that prescription opioids are readily marketed and sold online, both pre and post RHA [30-35]. This includes recent studies establishing a link between illicit online pharmacies that use social media channels (primarily Twitter) to market and directly sell opioids and other controlled substances (including fentanyls) [34-37]. Growing social media popularity among consumers and the fact that social media platforms are generally less regulated compared with other parts of the Web, such as indexed search engine results, may be driving these trends (eg, in 2011, Google was fined US $500 million by the US Department of Justice for knowingly allowing illegal ads for fraudulent pharmacies, including those selling controlled substances, and has since instituted certain policies for prevention [38]). The connection between illicit access and social media is particularly concerning given that many social media platforms are popular among young adults (a recent 2017 study reported that 30.85% of young adults used Twitter), a population at specific and increasing risk to prescription opioid addiction [39,40]. This clear risk to patient safety and the need for technology-based solutions has also recently caught the attention of the US Congress, with bipartisan letters from senators Chuck Grassley and Diane Feinstein sent to Google, Microsoft, Yahoo!, and Pinterest on February 15, 2018, warning them of the how their platforms are facilitating the online sale of illicit narcotics [41].

Hence, recognizing the need for innovative solutions leveraging advances in infoveillance, big data, machine learning, and Web forensic analysis, our team developed a method to detect marketing and sale of controlled substances via Twitter by online sellers. The main component of our solution was its use and establishing the viability of a proof-of-concept protocol for an unsupervised machine-learning algorithm to detect illicit online opioid seller tweets. We also created a prototype wireframe of a Web application to detect, classify, and report results for potential use by stakeholders such as the DEA, FDA, pharmaceutical manufacturers, and consumer patient safety groups.

## Methods

### Overview

The analysis for this study was conducted in two distinct phases including (1) Code-a-Thon challenge assessment and (2) “big data” analysis using machine learning of a Twitter dataset. We describe the design of our prototype Web application solution with a wireframe demo in the Results and Discussion section. The first phase involved coordinating with one of the coauthors of this manuscript, who is currently a CDC Entrepreneur in Residence, to scope out an appropriate challenge problem that fit the specific objectives and appropriate track of the Code-a-Thon (ie, addressing an under recognized threat in illegal opioid supply and access), identify the relevant datasets needed to address the challenge (ie, collecting a Twitter dataset associated with opioids precompetition), and organize team registration and logistics associated with Code-a-Thon participation. The second phase comprised data analysis conducted during the Code-a-Thon as described below. As this study involved the collection and analysis of existing publicly available data, it did not require institutional review board approval, nor was that required for participation in the Code-a-Thon.

### Data Collection and Analysis

After determining the challenge problem in partnership with CDC, we proceeded to collect messages (ie, tweets) published on Twitter over a period of approximately 20 days from November 15, 2017 to December 5, 2017 (the day before the start of the Code-a-Thon). The public streaming application programming interface (API) available from Twitter was used with certain preselected keywords that were a combination of International Nonproprietary Names and brand names of commonly abused opioids. Our final keyword list contained the terms codeine, fentanyl, hydrocodone, oxycodone, Oxycontin, Percocet, and Vicodin.

Upon commencement of the competition, we used a machine learning–based protocol to isolate word groupings associated with tweets that mentioned marketing and purported sale of prescription opioid drugs as has been carried out in prior studies by the first and second author [34,35,37]. To identify relevant tweets related to our challenge problem (ie, “signal” data) in large volumes of Twitter data (in the hundreds of thousands) vs nonrelevant data (ie, “noise” that contains opioid-related keywords but do not relate to online promotion or sale), the application of machine learning is critical to achieve scale and comprehensive analysis in a reasonable time frame compared with approaches solely using manual annotation by human coders.

**Figure 1:**
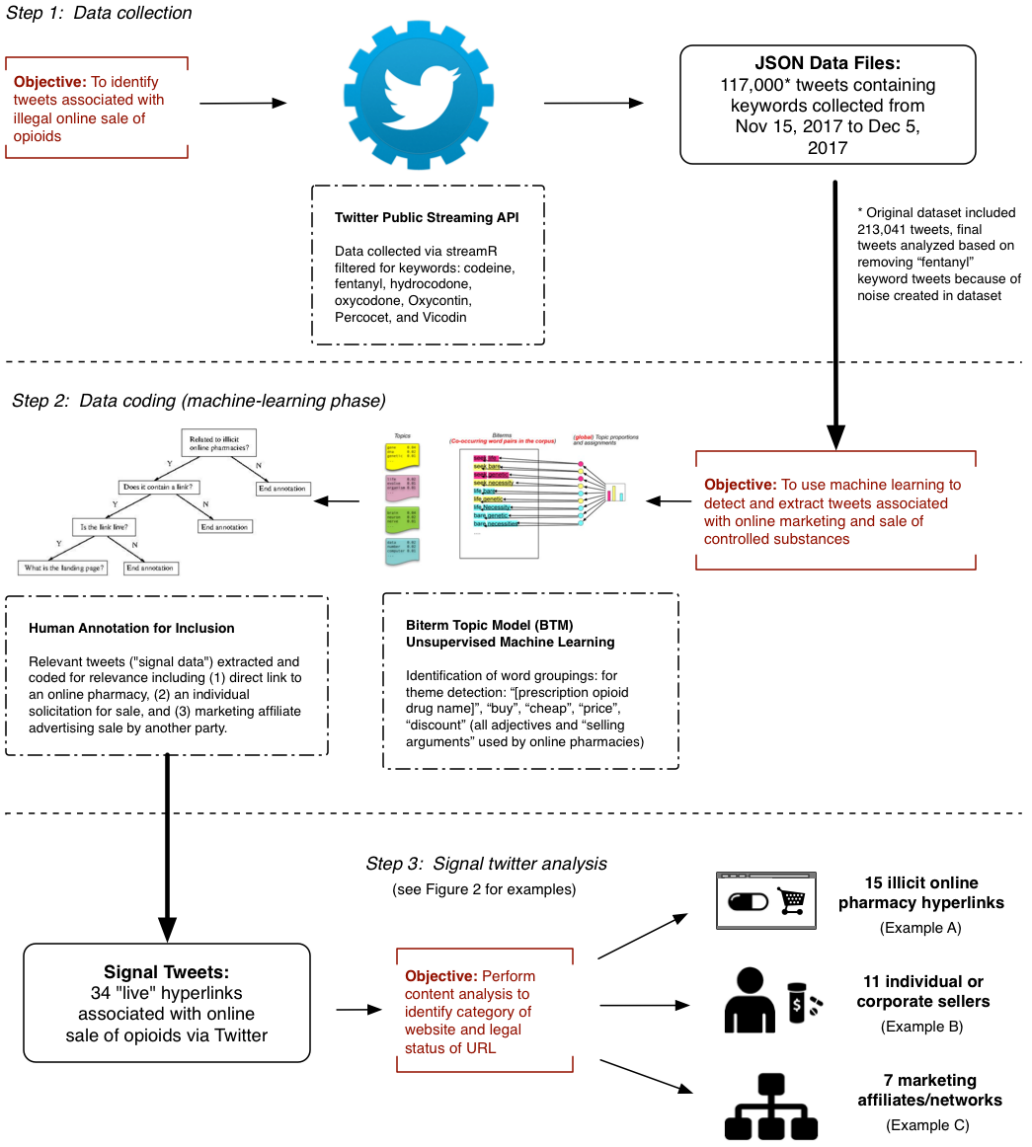
Summary of study methodology. API: application programming interface.

Specifically, unsupervised methods such as topic models prove to be useful in obtaining a summary of the underlying themes present in large text corpora. As we wanted to use methods that are able to capitalize on all the volume of our data collection, we chose unsupervised topic modeling–based methods over other approaches, such as those primarily based on analyzing hashtag co-occurrences [42]. We used a model called the biterm topic model (BTM) designed to detect themes and patterns in corpora of short texts (such as tweets), which we have previously used to examine prescription drug abuse behavior and online marketing and access (see Figure 1 for summary of this methodology) [35,43]. BTM was chosen because it is specifically designed to work in scenarios where the length of the documents or messages are short.

BTM (in its “learning” phase) first detected a preconfigured number of themes from the filtered dataset of tweets containing prescription opioid keywords. This produced a set of topics (or word groupings) that are thematic summaries of the contents of the entire set of tweets. The resulting word groupings are used to inform the next steps in the methodology—which are either identification of themes to detect signal associated with illegal marketing and sales of prescription opioids and to eliminate noise (discarding tweets that are deemed as “noisy” to isolate signal tweets) and then reapplying BTM on the smaller subset that has been filtered for noise until thematic saturation is achieved.

However, to ensure that BTM was the most appropriate method for this study, we also performed experiments with three other topic models: (1) Latent Dirichlet allocation, (2) Nonnegative Matrix Factorization, (3) and Kernel k-means. After each run of each model, we calculated both the cluster purity and perplexity scores for each of the topics and obtained the average across all clusters. The cluster purity measures how tightly knit each cluster of documents is, and hence, a higher purity is better. The perplexity measures how good a language model is at predicting words with a lower perplexity score being an indication of a good language model. On the basis of these tests, BTM scored higher across both metrics for different cluster numbers. Hence, we felt confident that using BTM for this particular data source had high performance compared with other topic models.

Once the learning phase of BTM produced a set of themes (or word groupings), each of the themes was manually annotated to identify “signal” themes clearly associated with prescription opioid marketing, distribution, and sale. Those themes that were marked as relevant (eg, a theme that would typically marked as being “relevant” is one with a combination of words including “[prescription opioid drug name],” “buy,” “cheap,” “price,” and “discount,” where these adjectives and “selling arguments” are identified as used by online sellers) were then extracted for further analysis to identify specific characteristics of the seller or marketplace by retrieving the tweets that were highly correlated with them using what is called the “inference” phase of BTM [44]. Within this subset of tweets, any false positives were first manually eliminated by two of our coauthors (TM and EK) by discarding those tweets whose content did not have a clear indication that the tweet was about the sale or promotion of a prescription opioid.

For example, as we narrowly focus on tweets purportedly offering online sale of opioids, only those tweets with hyperlinks contained in the message of the tweet or other contact information were considered. This produced a narrower group of tweets that contained hyperlinks to external websites or contract information for analysis. Hyperlinks were further manually coded to determine if the link was still active (ie, still redirecting to an external website vs a “dead” link that failed to redirect to a working website or produced an error code), whether it marketed or purported to directly sell an opioid product, and classified according to the nature and type of seller (eg, illicit online pharmacy, individual seller, or marketing affiliate) [24]. TM and EK coded for false positive tweets and the characteristics of links or websites independently and achieved a high intercoder reliability for results (*k=* 0.94). Discrepancies were resolved through reevaluation and consensus.

For all “signal tweets” that were specifically categorized as illegal online pharmacies, we also cross-referenced the URLs of these websites with LegitScript’s external database that includes a legal classification. LegitScript legal classification is based on its own assessment of whether the website is (1) “rogue”: vendor engaged in illegal, unsafe, or misleading activity, (2) “unapproved”: vendor with a problem of regulatory compliance or risk in one or more jurisdictions, (3) “unverified”: not subject to LegitScript review or monitoring, or (4) “legitimate”: passed LegitScript certification criteria [34,45]. LegitScript classification queries offer another layer of verification regarding an online pharmacy’s legal status and can help to confirm that the sites present high risk to consumers. We also reviewed WHOIS data to determine the internet protocol (IP) address and registered owner location for links classified as online pharmacies.

## Results

### Main Results

We collected data for approximately 20 days, resulting in a total of 213,041 tweets used for our analysis. The number of preconfigured themes was set to 100 during our BTM learning phase. During our first run of BTM on the entire dataset, we observed that a significant number of the word groupings were related to fentanyl, all of which were news-related themes (see examples in Table 2). This can be attributed to the high volume of news events surrounding illicit fentanyl before the time of the competition during the data collection phase. News essentially adds noise to the dataset (ie, it does not contain conversations related to online sales)—hence, we discarded fentanyl from the data and were left with approximately 117K tweets. Applying BTM on this smaller dataset gave clear signals of certain selling argument word groupings with words such as “buy,” “online,” “cheap,” “free,” “shipping,” and the name of a prescription opioid. This demonstrates that fentanyl-related tweets added noise to the dataset by suppressing signal data (see examples in Table 3).

**Table 2:** Some example themes that were obtained from the first round of biterm topic model (BTM).

Example theme	Word groupings
Example theme—1	fentanyl, deaths, dangers, nations, drugs, china, learn, surge, deals
Example theme—2	fentanyl, man, police, charges, faces, arrest, brockton, bust, Calgary
Example theme—3	fentanyl, overdose, suspected, teen, life, drugs, dead, death, Vancouver

**Table 3:** Some example themes that were obtained from the data after fentanyl-related tweets were removed (italics denotes relevant “signal” theme).

Example theme	Word groupings
Example theme—1	cannabis, legalization, respect, completely, opinions, varying, merits
Example theme—2	drug, war, opposition, benches, friends, conservative, political, dead, props
*Example theme—3*	*buy, online, pill, free, cheap, find, shipping, generic*

### Signal Twitter Data Analysis

Using the inference phase of BTM, we retrieved tweets that were most correlated and second most correlated to selling argument word groupings 0.32% (692/213,041). Of the total 692 tweets retrieved, 68.8% (476/692) contained hyperlinks. After manually coding tweets associated with these hyperlinks, we removed all duplicates (eg, retweets and identical tweets) and also any tweets with dead links. From these tweets, 23 unique Twitter user accounts were identified, with one account generating over 100K total tweets (containing both relevant and nonrelevant content) in 1 year, a characteristic common to social bot or spam accounts, a component of data analysis important in determining the source of content and whether it is biased [46]. All the other accounts examined had approximately 1000 tweets/year or lower and appeared to be human operated Twitter accounts. These user accounts generated a total of 34 live tweets with hyperlinks (followed by an estimated 1800 Twitter users during the duration of the data collection period). These tweets were then classified into one of three online seller categories: (1) an online pharmacy (defined as a website that purports to be an online pharmacy storefront, operates an e-commerce shopping cart where products can be checked out, paid and shipped directly to a consumer); (2) an individual seller or drug dealer (defined as a user offering the direct sale of prescription opioids via email, phone, or other direct contact solicitation); and (3) a marketing affiliates (defined as a website that hosts links to other websites that directly sell controlled substances; see Multimedia Appendix 1). Of the live hyperlinks coded at the Code-a-Thon, 44% (15/34) were online pharmacies, 32% (11/34) were individual sellers, 21% (7/34) were marketing affiliates, and 1 was purportedly from the Twitter handle of the darknet site AlphaBay (operated via the Tor network) linking to a reddit community page. The authenticity of the final result is questionable given that AlphaBay was shut down by law enforcement officials in July 2017.

The first category of 15 coded tweets consisted of 10 distinct live hyperlinks to illicit online pharmacies (see select examples in Multimedia Appendix 1, example A). All of these websites sold opioids with “no prescription” in combination with other drug products including other controlled substances (eg, barbiturates, Xanax, Ketamine, fentanyl patch, and codeine cough syrup), nonopioid prescription drugs therapeutic classes (eg, injectable steroids; antidepressants; weight loss drugs including Sibutramine, which has been removed from several markets; hormones; contraception; and erectile dysfunction drugs), recreational drugs (eg, cannabis), and some sites that also sold illicit drugs (eg, bath salts, MDMA, cocaine, heroin, and methamphetamines). One site purportedly accepted payment via cryptocurrencies bitcoin and ether (the cryptocurrency for the blockchain platform Ethereum). When cross-referencing for LegitScript status, 40% (6/15) were identified as “rogue,” with the remaining having no information available (ie, likely not detected or included in LegitScript’s database). Some online pharmacy tweets also used interesting selling arguments, including advertising Black Friday sales in reference to the day after Thanksgiving holiday in the United States, when retail discount sales are heavily marketed. Interestingly, when examining purported geographic location of IP addresses and website registered owners, the majority reported addresses in the United States.

The second category of 11 tweets appeared to originate from individual sellers using Twitter accounts that advertised the direct sale of opioids using hashtags (eg, #buy, #sell, #buypainmeds, #drugsforsale, #opioids, #painmeds, and #[controlled substance names]) and generally included contact information for the seller including an email address (in most cases a Google gmail account) and a phone number to call, text, or contact via the messaging application WhatsApp (phone numbers were mostly US 10-digit phone numbers, though phone numbers for UK country code +44 were also observed, indicating that these individuals may be located in these countries; see Multimedia Appendix 1, example B.) These tweets generally included pictures of prescription opioids and other drugs shown in an individual’s hand or displayed next to a pill bottle. Some Twitter individual accounts also linked to an external webpage (including WordPress domains), blogs, or online classified ad services. The sellers generally included in their tweets or external webpages a list of all drugs they offered to sell, often comprising a mix of controlled substances, other prescription drugs, and illicit drugs.

The third category consisted of seven tweets that included links to marketing affiliate websites and networks that were hosted on their own distinct domains or used other blog sites (eg, blogspot). These sites did not directly sell opioids but included information on how to purchase illegally from other sites and hosted hyperlinks that redirected traffic directly to an online pharmacy engaged in that activity (see Multimedia Appendix 1, example C). One of these sites included a frequently asked questions page that stated, “No, we are not sell any pills or medication. We are only provide medical information” and included several Web banners or advertisements to illicit online sellers, including one that used a fake banner claiming it was FDA approved.

### Web Application Wireframe Prototype Solution

**Figure 2:**
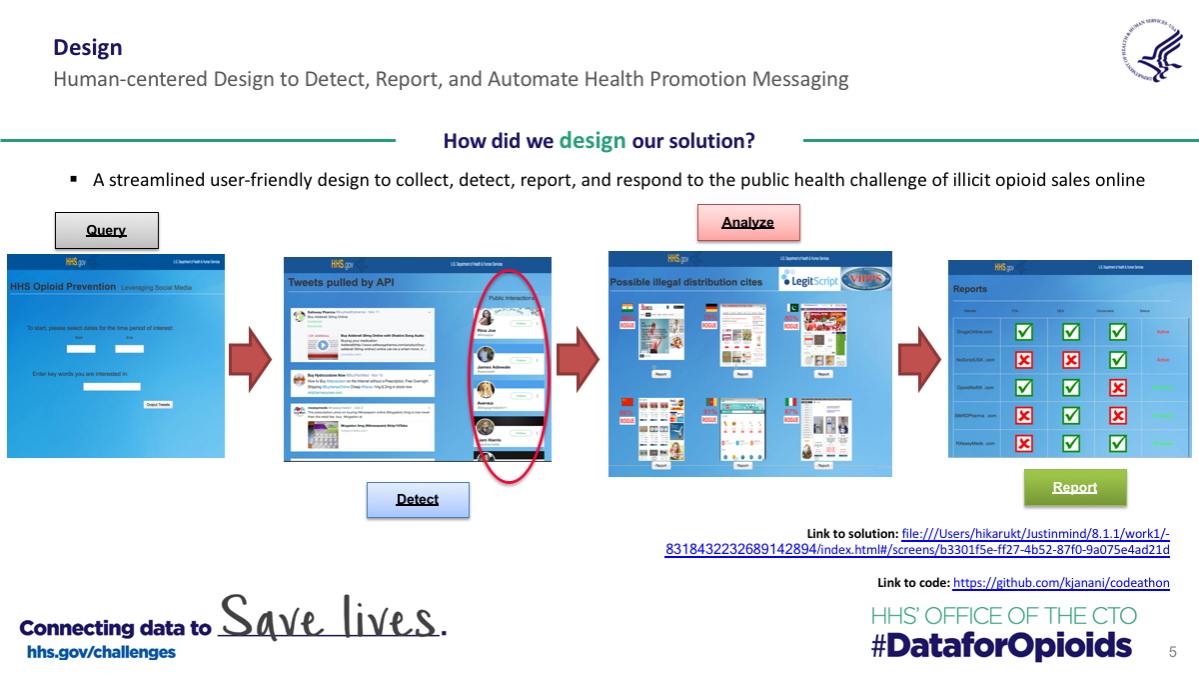
Design elements and screenshots of prototype wireframe solution.

In parallel with our Twitter data collection and analysis and to better demonstrate the potential real-world application of our BTM methodology to detect, classify, and report online pharmacies marketing the sale of controlled substances, we developed a wire frame solution using the prototyping tool for Web and mobile apps, Justinmind [47]. We based our conceptual model of our Web app solution on simplicity, streamlined navigation, and user interaction with a task focus. The Web app wireframe was designed to implement three primary functions: (1) data collection and detection of tweets marketing the sale of opioids online using BTM assisted by human interpretation, (2) HTML classification of websites to determine if they are illicit online pharmacies, and (3) automating a script to report to the FDA and DEA about detection of illicit online pharmacy for further regulatory action (see Figure 2 with screenshots of the justinmind wireframe solution developed and as presented during the Code-a-Thon). Targeted end users for the wireframe solution, which could operate as a hosted Web application or software, include pharmaceutical manufacturers, government agencies and regulators (FDA, DEA, and state Boards of Pharmacy), nongovernmental groups (NABP), consumer advocacy groups (the Alliance for Safe Online Pharmacies, ASOP, and the Center for Safe Internet Pharmacies), and potentially a modified version for the public to report suspect websites.

The first component of this solution included a webpage with a query function that included a date range for data collection and the ability to enter prescription opioid keywords for filtering of tweets from the public streaming API. The second page exports tweets filtered for selected keywords and then specifically outputs signal tweets highly correlated with selling arguments associated with online pharmacies. This step could also include an output of Twitter handles or accounts that have interacted with this high-risk content (eg, Twitter users that are followers, have retweeted, or favorited identified signal tweets) for possible targeted countermarketing, health communication regarding potential risk, and also generate data for potential social network analysis. The third page would then output screenshots of hyperlinks associated with signal tweets for human inspection, while also reporting the IP geographic location of the website (using WHOIS data) and the LegitScript legal classification of the URL if available (LegitScript operates a fee-based API for their information). This page would also include a “report” button under each website screen capture. User selection of violating websites would generate a final page detailing what URLs had been reported to the FDA and DEA using an automated script that would fill in the necessary information fields on the FDA’s “Reporting Unlawful Sales of Medical Products on the internet” webpage [48] and the DEA’s “Report Submission Form for Suspected Unlawful Sales of Pharmaceutical Drugs on the internet [49]. This prototype was presented during our finalist presentation at the Code-a-Thon, but because of time considerations, was only a click-thru but not fully functioning demo. However, illicit online pharmacy website results generated in this study were reported to both the DEA and FDA by manually filling out the online reporting tools. Once this information is reported, DEA and FDA exercise their own discretion on how to pursue enforcement against potential violations of federal law.

## Discussion

### Principal Findings

Similar to prior studies that have examined the use of Twitter by illicit online sellers to market and sell prescription opioids, our primary finding indicates that the overall volume of tweets directly engaged in this illegal activity is relatively low compared with the entire corpus of tweets collected that contained common opioid substance abuse product keywords [34,35,37,43]. This indicates that though this activity presents clear risk to the consumer and could contribute to diversion of opioids via convenient online access, the occurrence of these tweets are not widespread compared with other more commonly occurring conversations, including news reports about the opioid crisis, tweets about opioid abuse behavior self-reported by users, and other content that includes opioid keywords but which we would classify as noise per the aims of this study. However, some interesting results emanating from this study also provide us with key information on emerging trends regarding social media–enabled opioid abuse and methodological considerations when attempting to identify this type of content.

First, a major strength of this study compared with prior studies that conducted analysis on larger volumes of data was the short duration and immediate processing of the data collection and analysis (data collection was over a period of 2 weeks and immediately following it was the analysis over a 48-hour period). More specifically, previous studies involved a data collection and analysis process that was separated by more than 1 year [35,37,43,50]. By collecting and analyzing data immediately, fewer dead links where URLs were no longer active or website landing pages were no longer being used by owners were detected. Hence, our study demonstrates that a near real-time infoveillance approach, which collects and analyzes data over a short duration (eg, less than 30 days) can result in better detection and higher quality data related to illegal activities of online pharmacies and sellers. This approach could inure benefits to regulators and law enforcement officials, as Twitter accounts, illegal websites, and marketing affiliates could have their content taken down more quickly to mitigate potential exposure to consumers and accompanying patient safety risk. This would allow for proactive detection, particularly important given that illicit online pharmacies do not maintain a consistent presence on the internet and often change URLs frequently [24,51,52].

Immediate data coding and analysis also allowed us to detect our second category of tweets characterized as individuals who market and sell prescription opioids via open solicitations that had not been detected in prior studies. These digital drug dealers openly tweet that they can sell prescription opioids and other illicit substances directly to the public and that they can be contacted through a simple email or phone number. Oftentimes, they purport to validate availability of drugs offered by including a picture of their products included in the tweet and also use hashtags (#) to curate and target their marketing messages. These digital sellers sometimes represented themselves as individuals and in other cases as a company (primarily a name that represented itself as a pharmacy or pharmaceutical company). These results are alarming as they represent a potential new strategy where traditional “street” dealers may simply use Twitter in an effort to broadly market and extend their services to a wider and more diverse customer base that they would otherwise not have access to. The range of followers for these accounts varied, with observed ranges as few as 24 followers to as high as 989 followers, though generally these accounts were observed to have less than 100 followers. At the time of this writing, many of these accounts were still active on Twitter.

Additionally, a tweet we detected linked to a reddit community associated with the now defunct AlphaBay dark Web storefront leads us to unanswered questions and areas for future research. Specifically, this tweet raises the question of examining the interaction between social media, the open or clear Web accessible to the public, the dark Web accessed by clients such as tor or Onion, and illegal access to opioids. Other studies examining popular dark Web store fronts have shown that controlled substances and illicit drugs are actively sold on these sites to both consumers and potentially at wholesale to other distributors or dealers [53-55]. Hence, future studies should expressly examine the interaction between these different online ecosystems to determine their role in drug diversion and consumer purchasing.

Finally, we note that in addition to certain limitations, this study has some inherent weaknesses given that it examined a single social media platform: Twitter. According to the Pew Research Center, Twitter ranks at only #7 out of 8 popular social media platforms, with approximately 24% of US adult user share (compared with #1 YouTube at 73% and #2 Facebook at 68%), though use increases to 45% among users aged 18 to 24 year [56]. Hence, to get a more complete view of how controlled substances are marketed and sold to consumers online, future studies adopting machine-learning approaches used here for Twitter combined with the use of other methods, including deep learning (primarily for image recognition) and multiple modalities (such as simultaneously looking at additional data features), will need to be tailored to the types of data and user interactions occurring on other social media platforms (such as Instagram and Facebook) [57]. Additionally, lessons from prior studies that have examined how the internet has been used to circumvent the ban on purchasing illicit substances and how it has been used by minors to purchase alcohol and tobacco will also be informative to future technology-based and regulatory efforts against this illegal online opioid activity [58-63].

### Limitations

There are certain limitations associated with the study results generated and as reported in the Code-a-thon. Specifically, our sample of tweets was filtered for a select group of commonly abused prescription opioid drugs using their International Nonproprietary Names and brand names. For example, we did not collect street or slang names of these drugs (eg, oxy, roxies, percs, and vikes), as our prior studies that have examined both types of keywords indicated that illicit online pharmacies do not use these nonspecific words for marketing or sales, and most uses of street drug names are user behavioral–related [43]. However, further confirmatory analysis is needed to validate that online pharmacies consistently do not use street name terms for selling purposes. Additionally, we first cleaned the dataset before analysis to exclude non-English language tweets, which may further limit the generalizability of our sample of tweets containing opioid keywords. Furthermore, there are other strategies outside of topic modeling that can be used to learn about topical dynamics and trends in social media data, such as examining word and hashtag co-occurrences [42,64]. We chose not to use hashtag analysis as only one percent of all tweets in our dataset had hashtags in the content and less than one percent of signal tweets relevant to illicit online pharmacies used hashtags. For signal data that either included a direct hyperlink to an online pharmacy or marketing affiliates that redirected to live websites, content analysis was reviewed at a specific point of time after the tweets were collected and analyzed using BTM. Though tweets were coded right after the data collection was completed, it is possible that the content residing on hyperlinked content and/or the online pharmacy’s website or domain may have changed from the exact date of data collection as websites often update and change content. Additionally, our exclusion of “fentanyl” from our analysis may have removed content related to the illicit online sale of fentanyls via online pharmacies, user forums, and individual drug sellers but was necessary to further refine our results to detect signal tweets with clear selling argument word topic groups. A recent study by the first and second author conducted on an older 2015 fentanyl Twitter dataset can inform future analysis examining this drug class specifically [37]. The validity of WHOIS geographic data for online pharmacies reviewed is also unclear. Though many online pharmacies listed an IP address or registered owner address in the United States, their actual server location and/or physical business location or registration could be falsely entered or masked by a privacy internet service provider company. Finally, we note that the RHA and DEA explicitly have rules and regulations that make it illegal to purchase controlled substances online and have no clarifying guidance or exemptions in relation to conducting test purchases for research purposes. As researchers reside in US jurisdiction, we were unable to actually purchase controlled substances from online pharmacies and test them for authenticity.

### Conclusions

This study and our participation in the HHS Code-a-Thon established the viability of an important methodology to detect illicit online sales of opioids that are marketed via Twitter. Importantly, the machine-learning approach, which represents the core technology for our proposed solution and proof-of-concept deployed during the Code-a-Thon, is scalable and can be done relatively quickly following big data collection. This allows us to more rapidly detect illicit online sellers and classify their marketing characteristics. In fact, the vast majority of online sellers detected in this study remain active on social media and the Web at the time of this writing. Though the machine-learning component of this study is relatively mature, with testing of this algorithm and approach now published in four separate studies, the translation of the innovation to an easy-to-use, accessible, and largely automated solution is still at an early stage [34,35,37]. Though our prototype wireframe demonstrates the potential extension of the BTM machine-learning algorithm into a Web application that could be used by key stakeholders such as the FDA and DEA, it does not have the functionality or integration of different data sources to be considered a minimally viable product (MVP). To take this next step, funding with the primary aim of translating this research into MVP phase and eventual production and scale-up is needed (such as our recent award of an NIH NIDA 2017 “Start a SUD Startup” challenge, which provides small awards for startups related to substance abuse disorders with the aim of transitioning companies to a successful NIDA Small Business Innovation Research grant; see [65] for a recorded video discussing Code-a-Thon solution submitted as part of NIH NIDA 2017 “Start a SUD Startup” challenge grant). Additionally, we would need to automate data collection and backend analysis of Twitter data with integration via a Web application or software, while also developing solutions using natural processing language to automate classification of hyperlinks suspected as engaged in the sale of prescription opioids, techniques that have been explored in prior studies [23,66,67]. Finally, automated scripts that generate information needed for standardized reporting of results to the FDA and DEA via their online Web forms would also need to be developed. Despite these challenges, results from this study are useful and can inform regulators, law enforcement, public health officials, and the public about current and changing trends regarding supply, access, and distribution of illicit opioids. Technology, such as the big data and machine-learning approaches used in this study, will be critical components of any strategy to combat the opioid epidemic, an approach that HHS through its Code-a-Thon has begun to catalyze.

## Abbreviations

API: application programming interface
ASOP: Alliance for Safe Online Pharmacies
BTM: biterm topic model
CDC: Centers for Disease Control and Prevention
DEA: Drug Enforcement Agency
FDA: Food and Drug Administration
GAO: Government Accountability Office
HHS: US Department of Health and Human Services
IP: internet protocol
MVP: minimally viable product
NABP: National Association of Boards of Pharmacy
RHA: Ryan Haight Online Pharmacy Consumer Protection Act

## Appendix

Multimedia Appendix 1 Examples of illicit online pharmacies, individual sellers, and marketing affiliates (examples A, B, and C).
