# Supplementary figures and images for: Solution to Detect, Classify, and Report Illicit Online Marketing and Sales of Controlled Substances via Twitter: Using Machine Learning and Web Forensics to Combat Digital Opioid Access
Source: J Med Internet Res. 2018 Apr 27;20(4):e10029. doi: 10.2196/10029 (PMC5948414; doi:10.2196/10029)

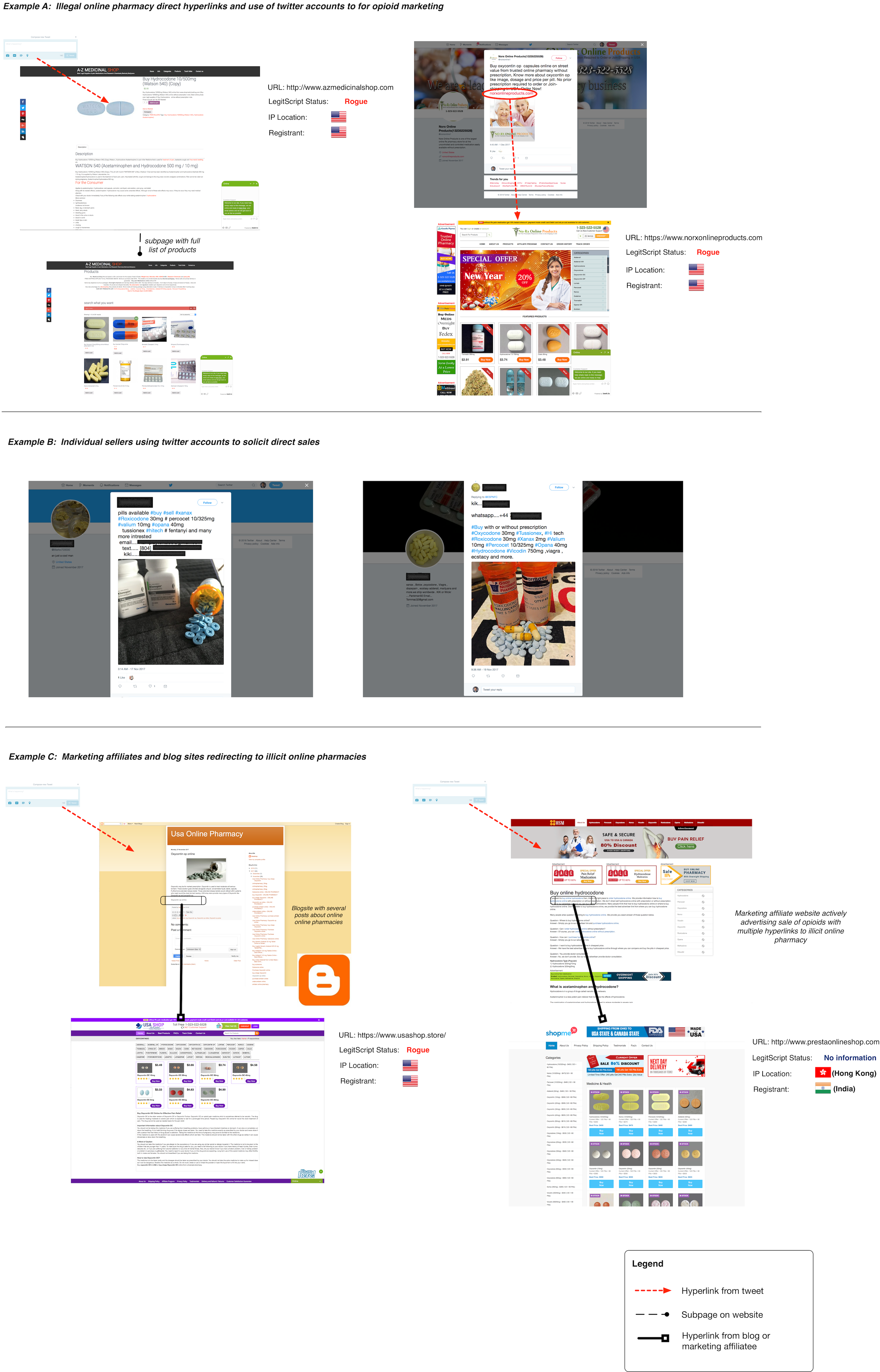

Supplement: Multimedia Appendix 1 [file jmir_v20i4e10029_app1.png]
